# Supplementary material for: De novo motor learning creates structure in neural activity that shapes adaptation
Source: Nat Commun. 2024 May 14;15:4084. doi: 10.1038/s41467-024-48008-7 (PMC11094149; doi:10.1038/s41467-024-48008-7)
Supplement: Supplementary file 1 — Supplementary Information [file 41467_2024_48008_MOESM1_ESM.pdf]

Supplementary Figures

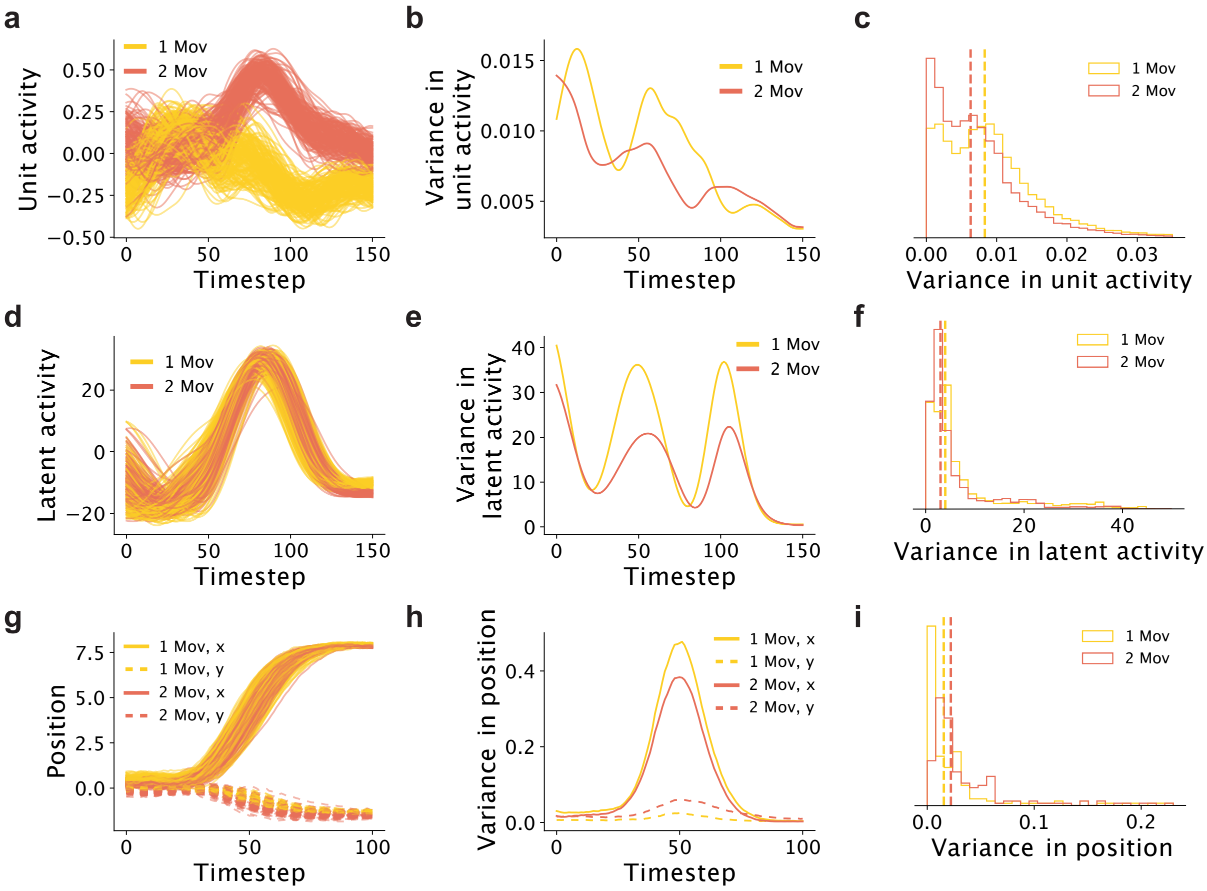

Supplementary Figure S1: Networks that can generate multiple movements produce more constrained neural dynamics. This figure presents additional data for Figure 2b–d, and compares different variables across networks with different repertoires producing the same common movement. **a**. Unit activity for one example unit for networks trained on one (yellow) or two (orange) movements. Networks had the same random seed. Traces, different trials. **b**. Variance in unit activity, calculated per timestep across all trials for example unit in Panel a. **c**. Distributions for variance in unit activity, pooled across all units for one example seed. Dashed lines, median. **d-f**. Same as Panels a-c but for the second dimension of the latent dynamics in Panels d-e and for all dimensions in Panel f. **g-i**. Same as Panels a-c but for the motor output. In Panels g-h: Solid line, position along the x axis; dotted line, position along the y axis.

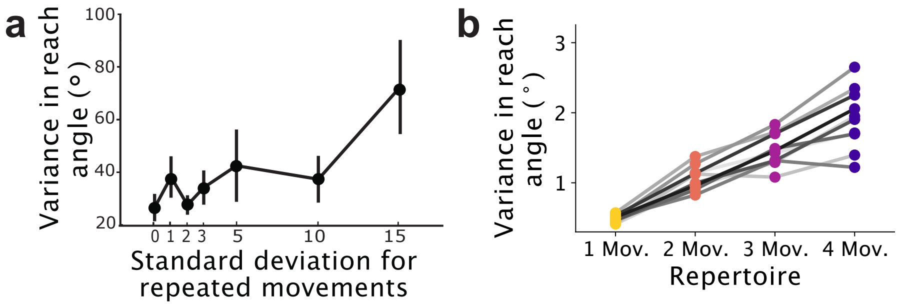

Supplementary Figure S2: Networks reproduce experimental results from Ref. 54. **a**. This study examined the variance in reach angles to a given target following repeated movements sampled from a normal distribution around the target with different standard deviations. Larger variance of known movements was correlated with larger variance in reach angle. Figure modified from Fig. 2a from Ref. 54. **b**. Networks initially trained on different repertoires were tested on one shared movement. Networks trained on larger repertoires had a larger variance of known movements, and this was also correlated with larger variance in reach angle.

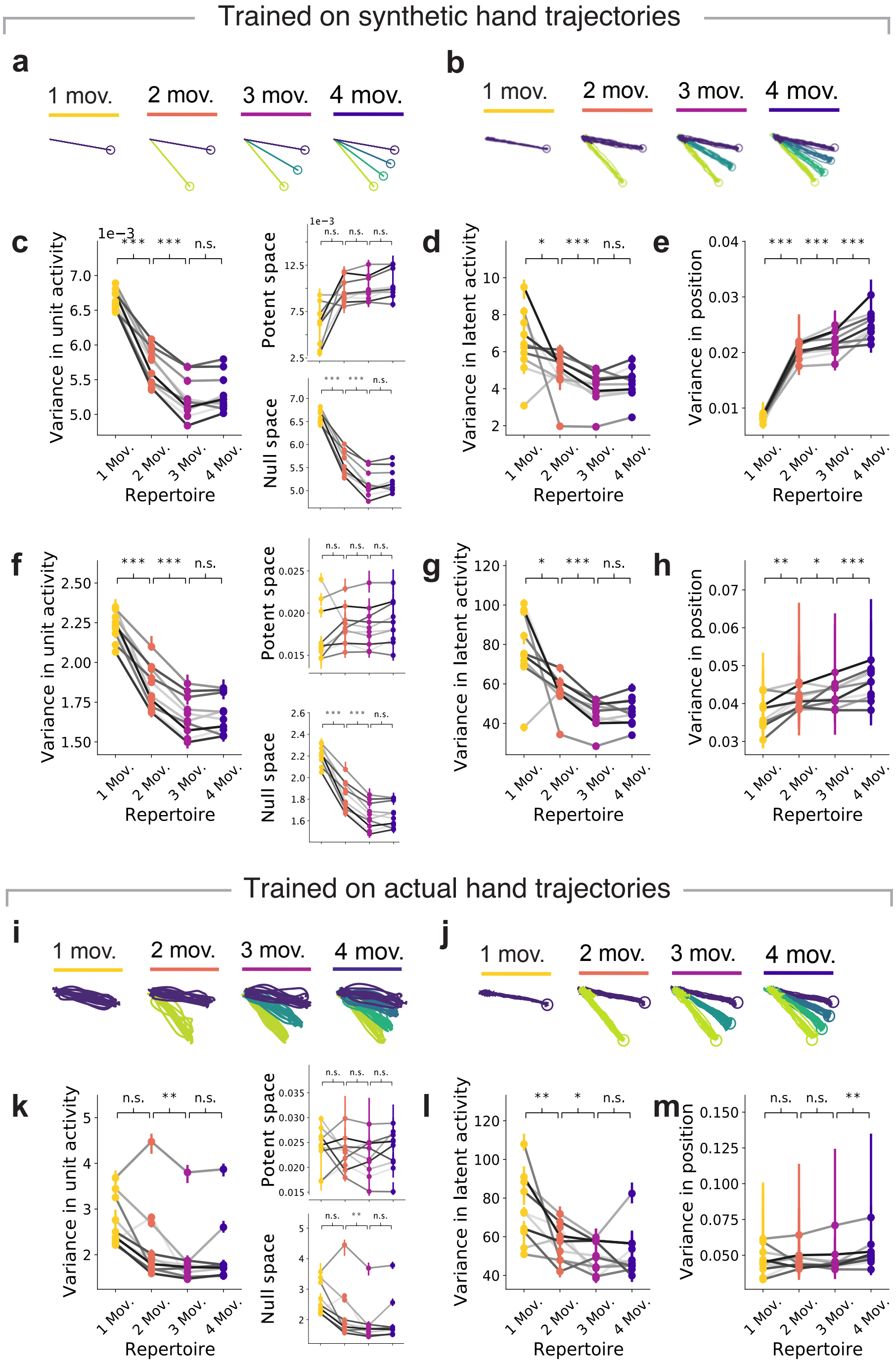

Supplementary Figure S3: Trends in variance remain robust for synthetic movements and different measures of variance. a-h. Networks were trained to produce motor output (**b**) based on simulated (**a**) rather than actual "hand trajectories" based on monkey movements. Simulated trajectories did not have trial-to-trial variability for a given movement. **c-e**. Same as Fig.2c–d but for networks trained on simulated hand trajectories. **f–g**. Same as Panels c–e but for total variance summed across all features (across all units for unit activity, latent dimensions for latent activity, and output dimensions for position) rather than the variance for each individual feature. Variance is still calculated for each timestep across trials. Note that the patterns remained the same for synthetic trajectories as when trained on actual hand trajectories, showing that constrained dynamics are not a byproduct of variability in the monkey movements they were trained on. **i–m**. Networks were trained to produce motor output (**i**) based on actual "hand trajectories" based on monkey movements (**j**). These are the

same simulations as in the main text. **k–m**. Same as Panels f–g but for networks trained on experimental trajectories. Note that the trends remain the same. While some trends are not as strong, they are likely due to masking from the variability inherent in experimental trajectories, since the trends remain strong for the networks trained on synthetic trajectories.

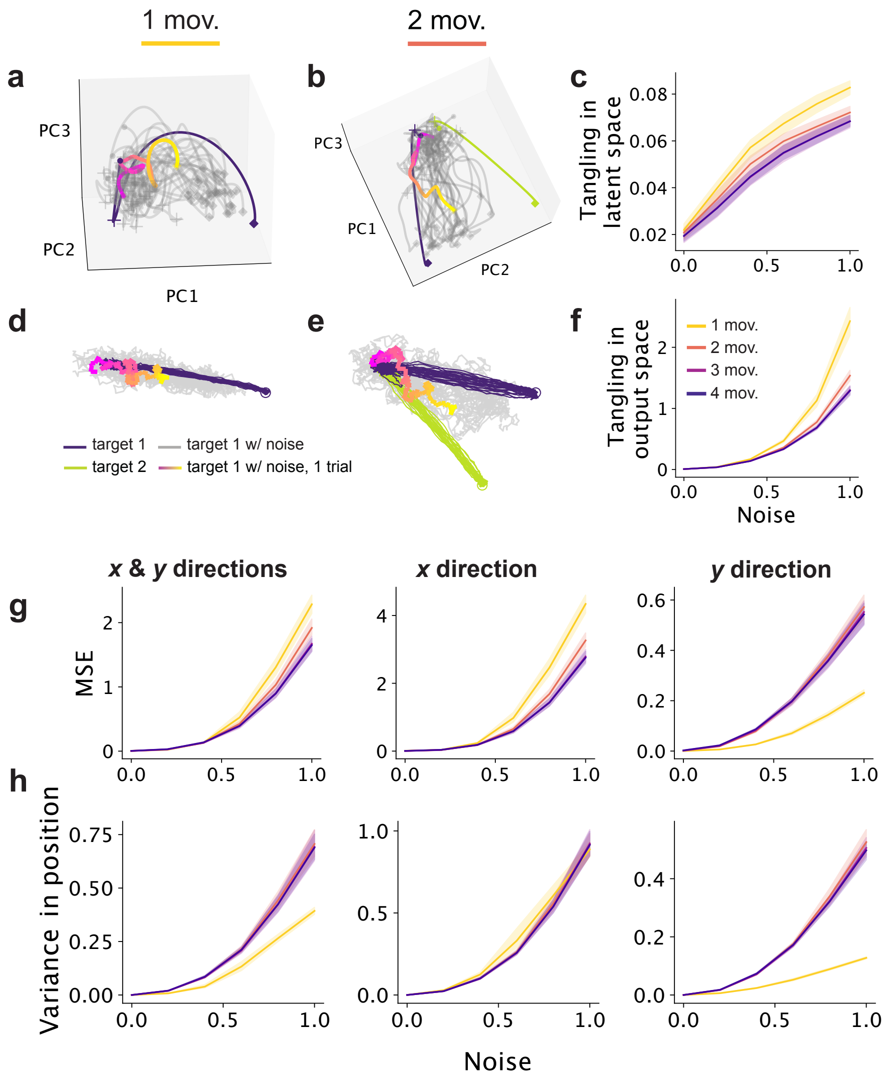

Supplementary Figure S4: Addition of noise shows underlying structure in multi-movement networks. Noise was applied to networks trained on synthetic trajectories without trial-to-trial variability to guarantee that differences would be due to different repertoires rather than different variability in the training data. **a**. Latent trajectories for an example single-movement network. Purple line, trial-averaged trajectory without increased noise added. Pink gradient and grey lines, trajectories for example trials when increased noise ($\eta = 1$) is added. Color gradient, time-course of the trial. **b**. Same as Panel a but for an example two-movement network. Noise is only added to the first movement. **c**. 90th-percentile tangling in the neural space[86], which quantifies how deterministic future states are from current states. **d-f**. Same as Panels a–c but for the motor output and output space. **g**. Mean-squared error (MSE) of output when noise of increasing magnitude is added to the neural activity. Line and shaded area, median and 95% confidence interval. Left: MSE for both x and y directions. Middle: MSE only for the x direction. Right: MSE only for the y direction. **h**. Same as Panel g but for the variance in position.

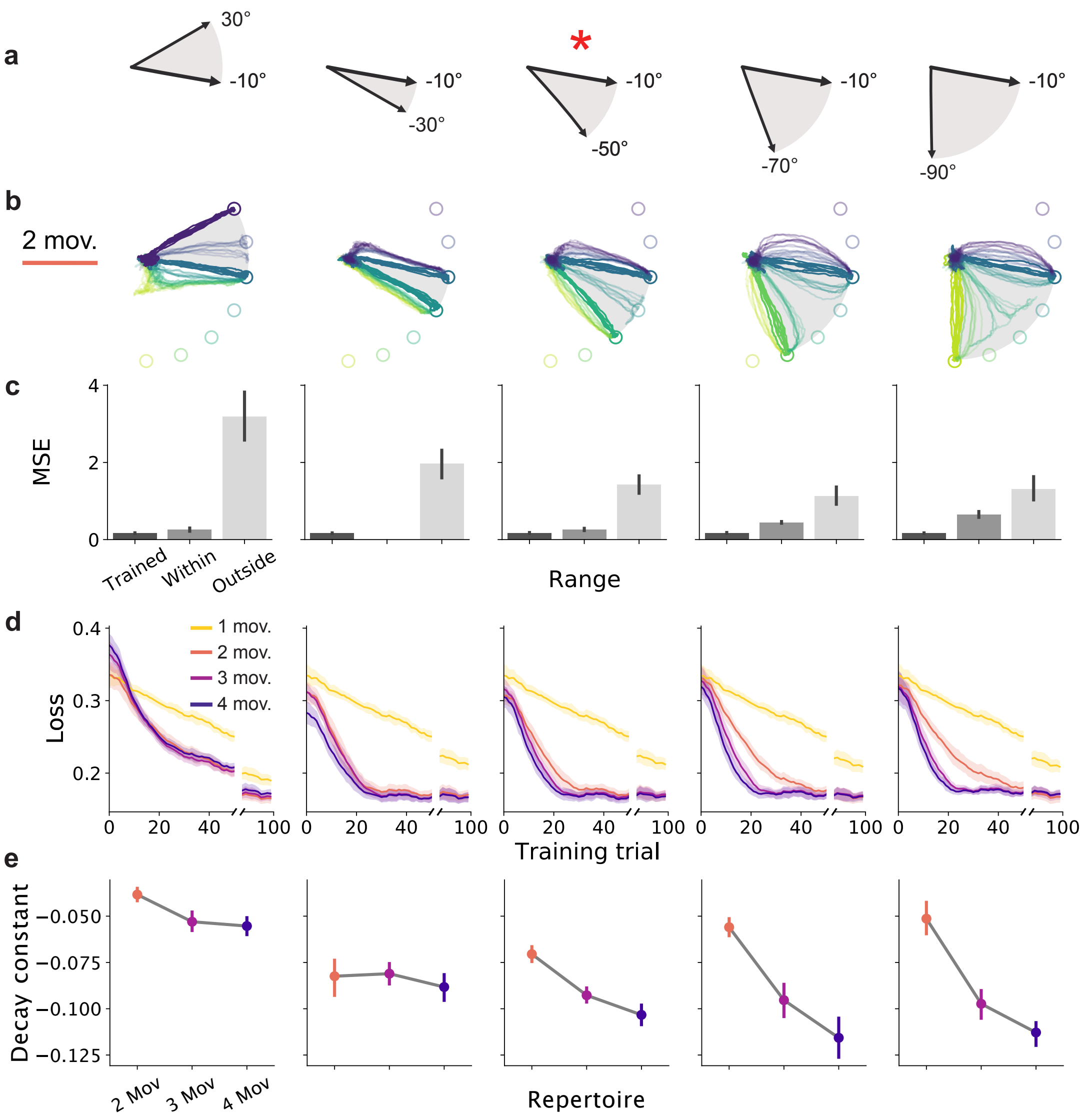

Supplementary Figure S5: Networks can generalize and adapt to perturbations that require movements within a learned range. **a**. Networks were trained on repertoires with movements that spanned different ranges (top). The range -10° to -50°, denoted by an red asterisk, was used for all simulations in the main text. **b-c**. Networks trained on two-movement repertoires in their respective ranges (i.e. a movement to -10° and a movement to -50° for the -10° to -50° range) were tested on target cues for movements equally spaced between 30° and -90° to assess whether they could generalize to movements they were not trained on. The target cues were chosen such that networks were tested on movements they knew ('Trained'), movements that were within the range of known movements ('Within'), and movements that were outside the range ('Outside'). **b**. Motor output for the target cues for each movement for a sample network. Colors, target cues; circles, targets for each movement. Colors for target cues that the networks have not been trained on have lower opacity. Grey backgrounds denote the range of known movements. **c**. Mean-squared error between the network output and target positions for previously 'Trained' movements, and movements 'Within' or 'Outside' the range of known movements. Note that MSE was lower for 'Within' movements, showing that the networks can generalize. **d**. Loss during adaptation training with counterclockwise VR perturbations of 10°. Traces and shaded areas, mean and 95% confidence interval across networks of different seeds. **e**. Decay constants for exponential curves fitted to the loss curves in Panel d. Circles and error bars, mean and 95% confidence interval with bootstrapping.

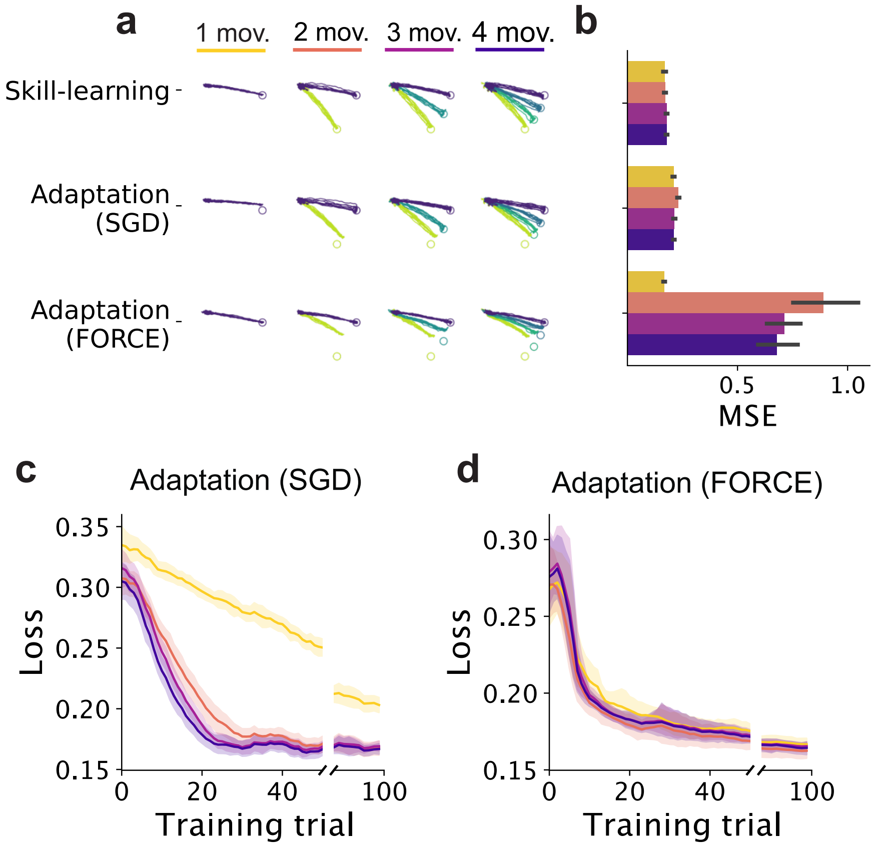

Supplementary Figure S6: Adaptation training with stochastic gradient descent (SGD) is better at overcoming catastrophic forgetting than FORCE. Following skill learning, networks were trained to adapt to counterclockwise VR perturbations of 10° on one movement, using either SGD or FORCE. Motor output (**a**) and MSE (**b**) for all movements in known repertoires following adaptation training. Note that networks trained with FORCE had worse 'catastrophic forgetting' (that is, forgetting of previously trained tasks when learning new tasks) of other movements that were not perturbed during adaptation training. Loss during adaptation training with SGD (**c**) and FORCE (**d**). Traces and shaded areas, mean and 95% confidence intervals across networks of different seeds.

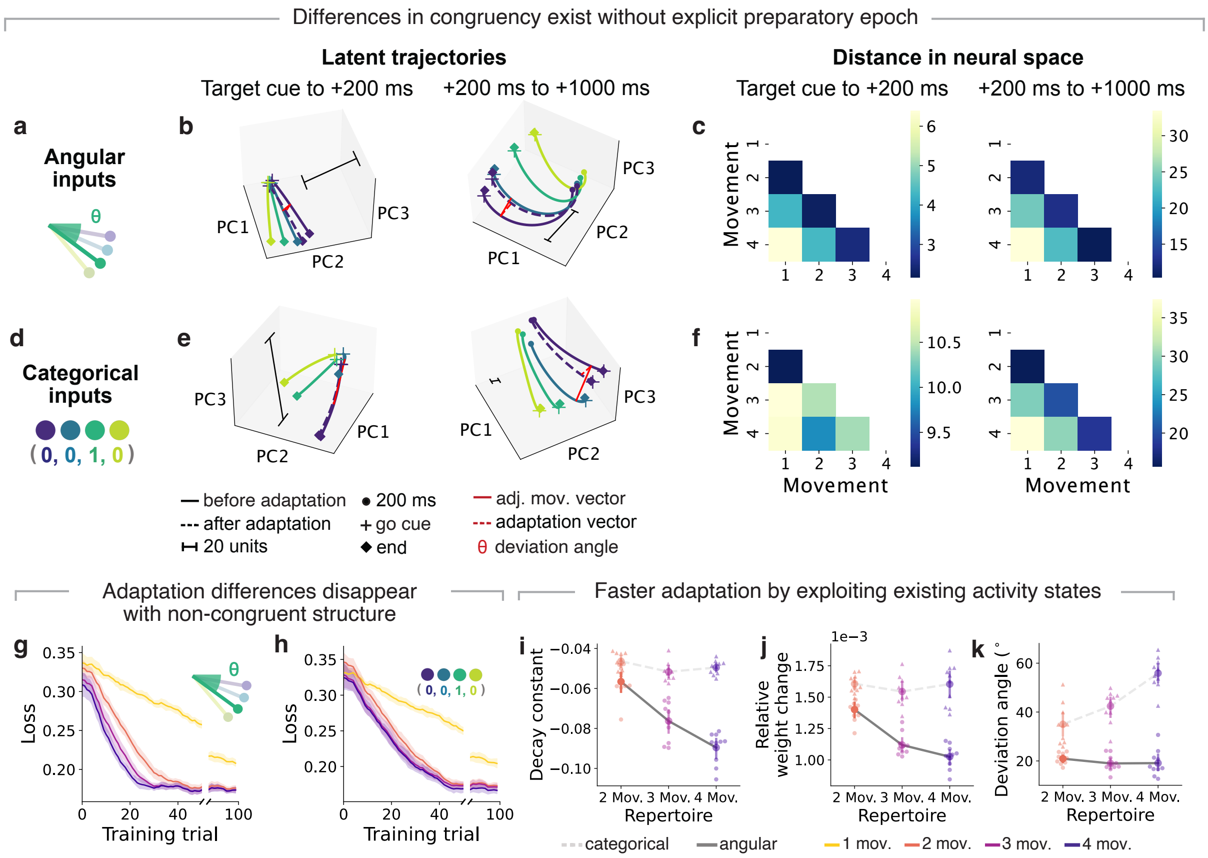

Supplementary Figure S7: Explicit preparatory epochs are not necessary for differences in adaptation and structure. Networks were trained to produce movements without an explicit preparatory epoch. That is, networks started movement at the target cue rather than waiting for a go cue. Without an explicit preparatory epoch, networks still had preparatory-like activity in the first 200 ms after the target cue and execution-like activity for the next 800 ms after the target cue. **a–f**. Same measures as Figure 4a–f, but with the distances in neural space separated into the first 200 ms after the target cue and 200 to 1000 ms after the target cue. **g–k**. Same measures as Figure 4g-l.

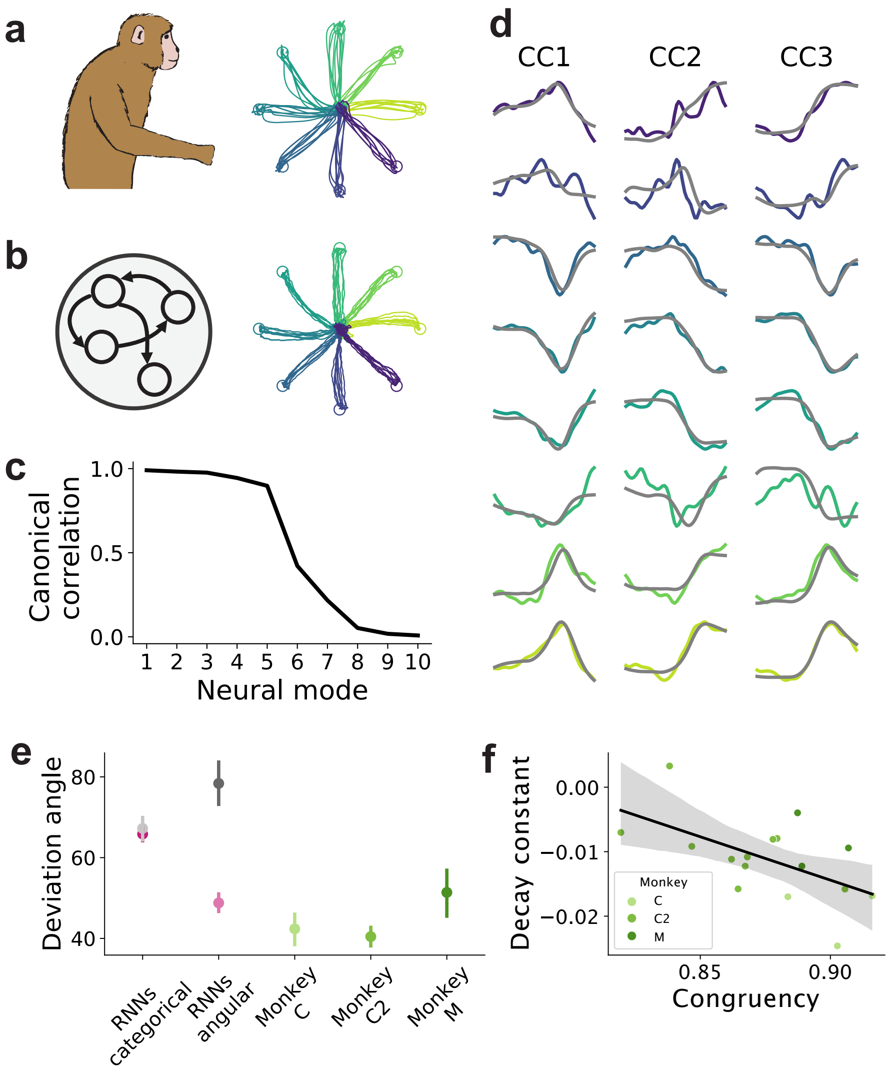

Supplementary Figure S8: Simulation results reflect experimental monkey data. **a**. Hand trajectories produced by a monkey performing an eight-target center-out reaching task. Data from Ref. 6 (pooled from all sessions for Monkey M, see Methods). **b**. Simulated motor output when RNNs were trained on the same center-out reaching task with angular inputs. **c** Canonical correlation values between experimental and simulated latent dynamics. **d**. Projections of the experimental (colored) and simulated (grey) latent dynamics onto the first three axes identified through canonical correlation analysis. **e-f**. RNNs were trained on the center-out reaching task with either one-hot encoded ('categorical') or angular inputs, and compared to monkeys trained on the same task (Monkey C, Monkey C2, and Monkey M). Data from Ref. 6. **e**. The 'deviation angle' (Figure 4k) was calculated and pooled for the latent activity of all targets before and after adaptation to a visuomotor rotation. Circles and error bars, median and 95% confidence intervals with bootstrapping. Grey, control with shuffled targets and time points. **f**. Congruency between angular input structure and neural space structure (see Methods) compared to the decay constants fit to learning curves based on the angular reach errors.

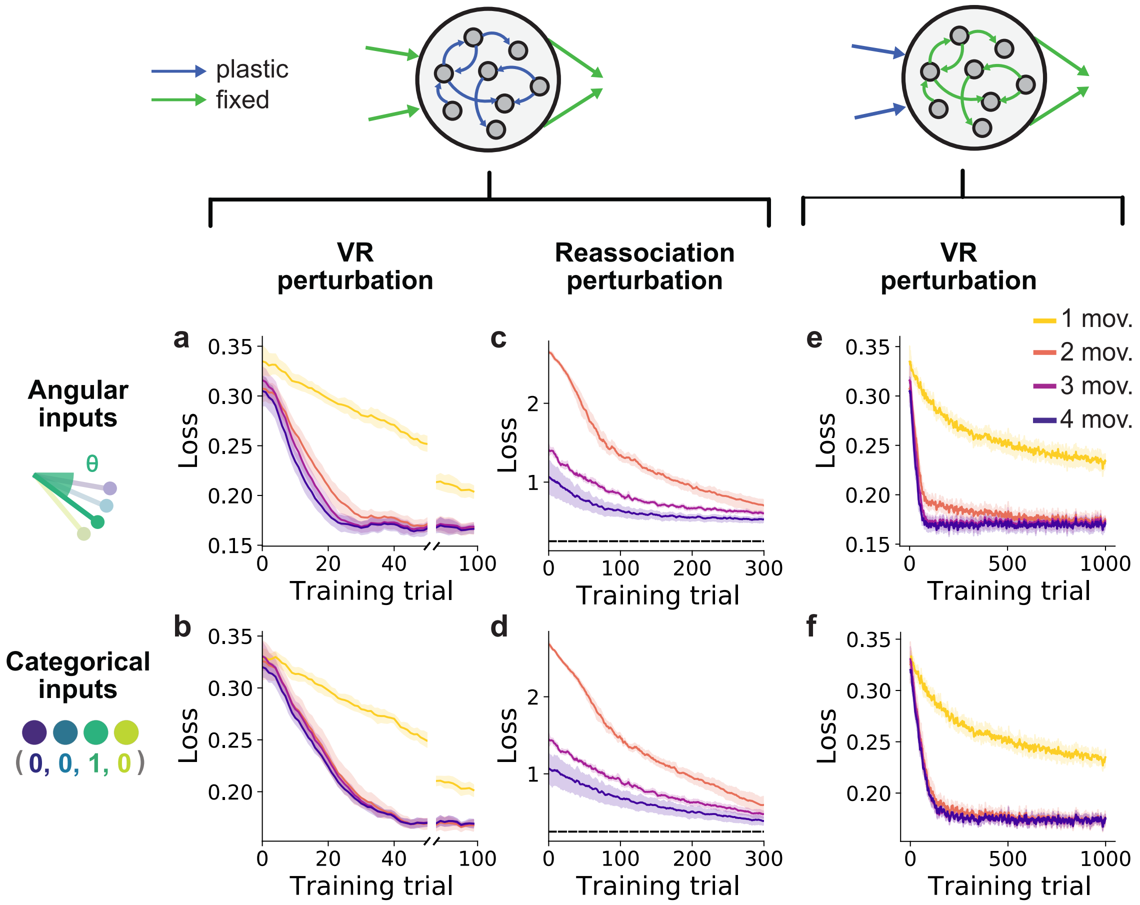

Supplementary Figure S9: Adaptation with fixed input or recurrent weights. Loss during adaptation training with 10° counterclockwise VR perturbations with fixed input weights for networks with angular (**a**) and categorical (**b**) inputs (for 100 training trials). Traces and shaded areas, smoothed mean and 95% confidence intervals across networks of different seeds. **c, d**. Same as Panel a, b but for reassociation perturbations (for 300 training trials). Note that the results from Figure 5a,d held when the input weights were frozen such that the networks could not simply rely on changing the input weights to counteract reassociation. **e, f**. Same as Panels a, b but with fixed recurrent weights (for 1000 training trials). Note that the patterns remain the same as in the main text, where networks were trained with plastic input and recurrent weights, although at longer timescales.

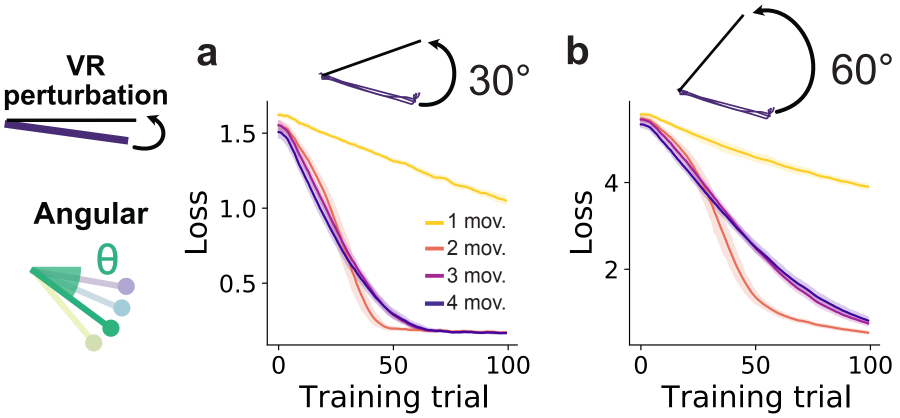

Supplementary Figure S10: Structure in neural space hinders adaptation when larger changes are required. Networks were given angular inputs and adapted to 'more challenging' VR perturbations than the 10°rotations examined thus far. **a**. Loss during adaptation training for a VR of 30°. **b**. Loss during adaptation training for a VR of 60°. For both perturbations, smaller multi-movement networks adapted more quickly than larger multi-movement networks, contrary to previous results under smaller perturbations of 10° (Figure 3d). Note that this effect occurred when the movement needed to counter the adaptation was both within (for the 30° VR) and outside the range (for the 60° VR) of the known movements.

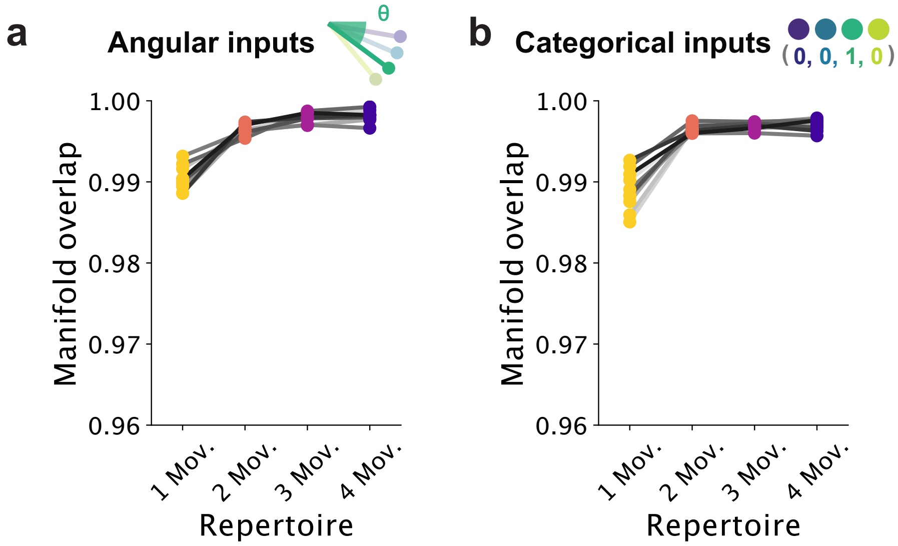

Supplementary Figure S11: Adaptation to visuomotor rotation perturbation is within-manifold. Manifold overlap (see Methods) for network activity between skill learning and adaptation to a VR perturbation of 10° for networks with angular (**a**) and categorical (**b**) inputs. Line colors denote different random seeds.

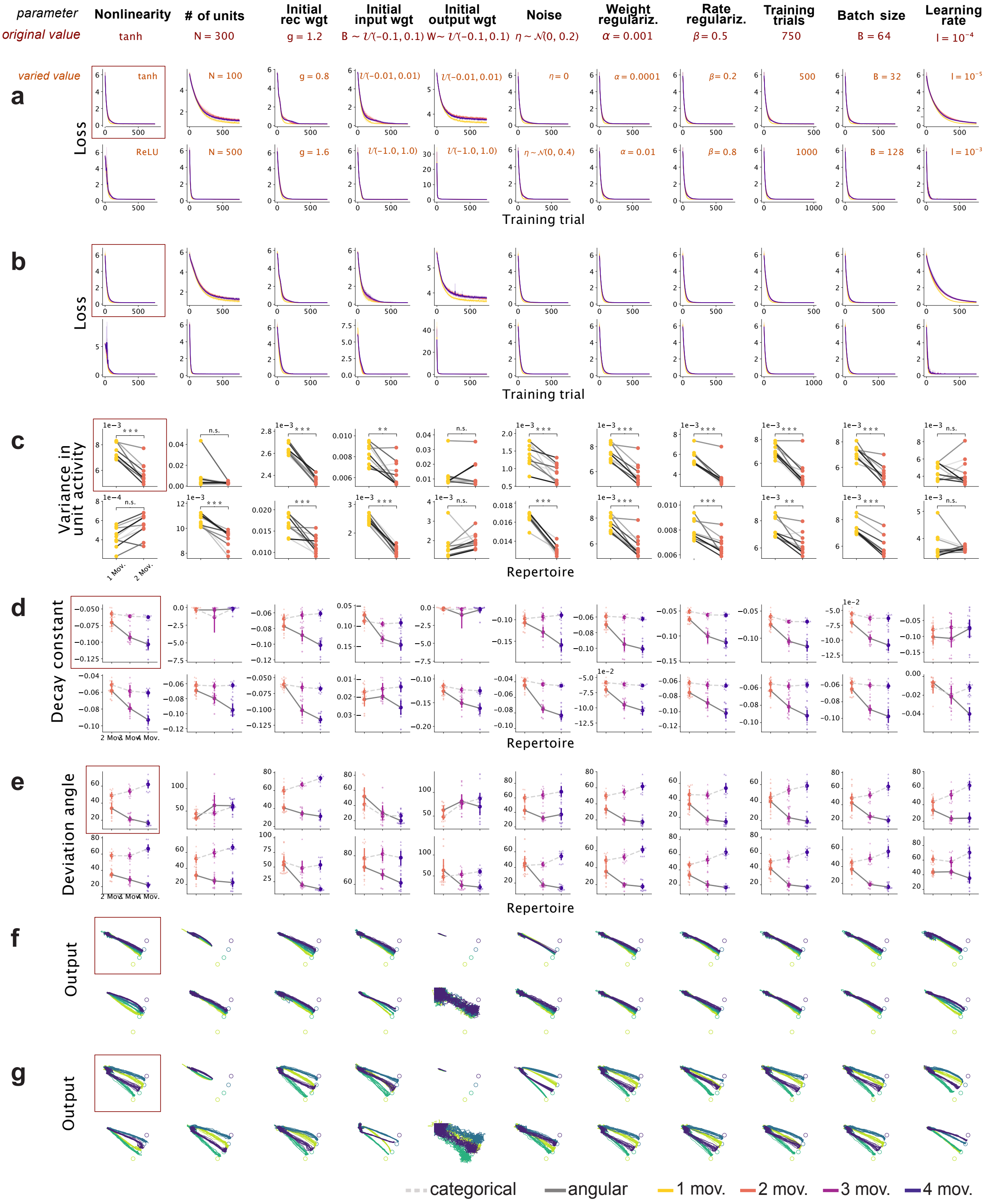

Supplementary Figure S12: Main results remain robust to changes in modeling parameters. Parameters in the neural network model were varied one at a time and simulations were run with these changes. The parameter values in red denote the original value used in the simulations in the main text. The red boxed plots denote the results for the simulations in the main text. The values in orange in (**a**) denote the changed values. The trends in the results were generally robust to changes in parameters. **a**. Loss curves during initial training for networks with angular inputs. Presented in Figure 3b. **b**. Loss curves during initial training for networks with categorical inputs. **c**. Variance in unit activity for angular input networks. Presented in Figure 3c. **d**. Decay constants for angular and categorical input networks. Presented in Figure 4i. **e**. Decay constants for angular and categorical input networks. Presented in Figure 4l. **f**.

Output for reassociation perturbation for angular input networks. Decay constants for angular and categorical input networks. Presented in Figure 5c. **g**. Output for reassociation perturbation for angular input networks. Decay constants for angular and categorical input networks. Presented in Figure 5f.
